# Spinal Cord Infarction Mimicking Acute Transverse Myelitis

**DOI:** 10.7759/cureus.1911

**Published:** 2017-12-06

**Authors:** Nilesh H Pawar, Ealing Loke, Derrick C Aw

**Affiliations:** 1 Department of General Medicine, Sengkang General Hospital, Sengkang Health, Singhealth

**Keywords:** spinal cord infarction, posterior spinal cord infarction, stroke, bilateral lower extremity weakness

## Abstract

Spinal cord infarction (SCI) is a rare type of stroke. The initial magnetic resonance imaging (MRI) is usually normal and can mimic the presentation of the acute transverse myelitis (ATM), acute inflammatory demyelinating polyneuropathy, and compressive myelopathies from neoplasm, epidural or subdural hematoma, or abscess. The aim of this report is to describe and discuss the case of a patient with SCI presenting as a diagnostic confusion with acute transverse myelitis. A 64-year-old male with a medical history of hypertension presented with an acute onset of urinary retention with lower limb weakness. Based on the initial MRI and evaluation, a diagnosis of acute transverse myelitis was made. Despite thorough evaluation, the etiology of transverse myelitis was undetermined. Hence, the MRI of the thoracic spine was repeated which showed patchier enhancements of the vertebral body with features suggestive of the spinal cord and vertebral body infarction. Thus, a repeat MRI is required to make an accurate diagnosis. The vertebral body is always involved and can be of diagnostic significance as it reflects the pathology of underlying blood supply.

## Introduction

Spinal cord infarction (SCI) is a rare condition and represents only 1% of all the strokes [1]. The blood supply to the spinal cord is derived from one anterior and two posterior spinal arteries [1]. Both of the posterior spinal arteries (PSAs) have extensive branches; as such, PSA infarction is very infrequent in comparison to the anterior spinal artery infarction [1]. The PSA infarction classically presents with unilateral involvement. Uncommonly, the territorial involvement may be bilateral, causing diagnostic confusion and delay [1]. The onset of symptoms in PSA infarction can be acute, subacute, and chronic [2]. As a result of its inconsistent clinical involvement, it can mimic other conditions like transverse myelitis, acute inflammatory demyelinating polyneuropathy and compressive myelopathies from neoplasm, epidural or subdural hematoma, or abscess [3]. Here, we present an interesting case of SCI, which mimicked acute transverse myelitis (ATM) and puzzled the clinicians.

## Case presentation

A 64-year-old Chinese male with the past medical history of hypertension presented with acute urinary retention associated with bilateral lower extremity weakness and numbness since he woke up in the morning. On examination, he was afebrile and the blood pressure measured 169/100 mm Hg. The clinical examination revealed normal mental function and cranial nerve examination. The motor system examination revealed decreased strength over both the lower extremities (the medical research council grade 3/5 at left hip joint, 4/5 at left knee joint, 3/5 on dorsiflexion of left ankle joint, 3/5 on the plantarflexion of left ankle joint, 3/5 at the right hip joint, 4/5 at the right knee joint, 4/5 on dorsiflexion of right ankle joint and 4/5 on the plantar flexion of right ankle joint). The lower limb deep tendon reflexes were exaggerated (bilateral knee-jerk and ankle jerks were 3+ and 2+ respectively) with bilateral upgoing plantar responses. The tone was spastic bilaterally. The position sensation and vibratory perception were impaired, and pinprick hypoesthesia was noted below the level of thoracic spine T11. The digital rectal examination revealed diminished anal sphincter tone and perianal hypoaesthesia. There was no upper limb pathology. The rest of the physical examination was noncontributory.

**Figure 1:**
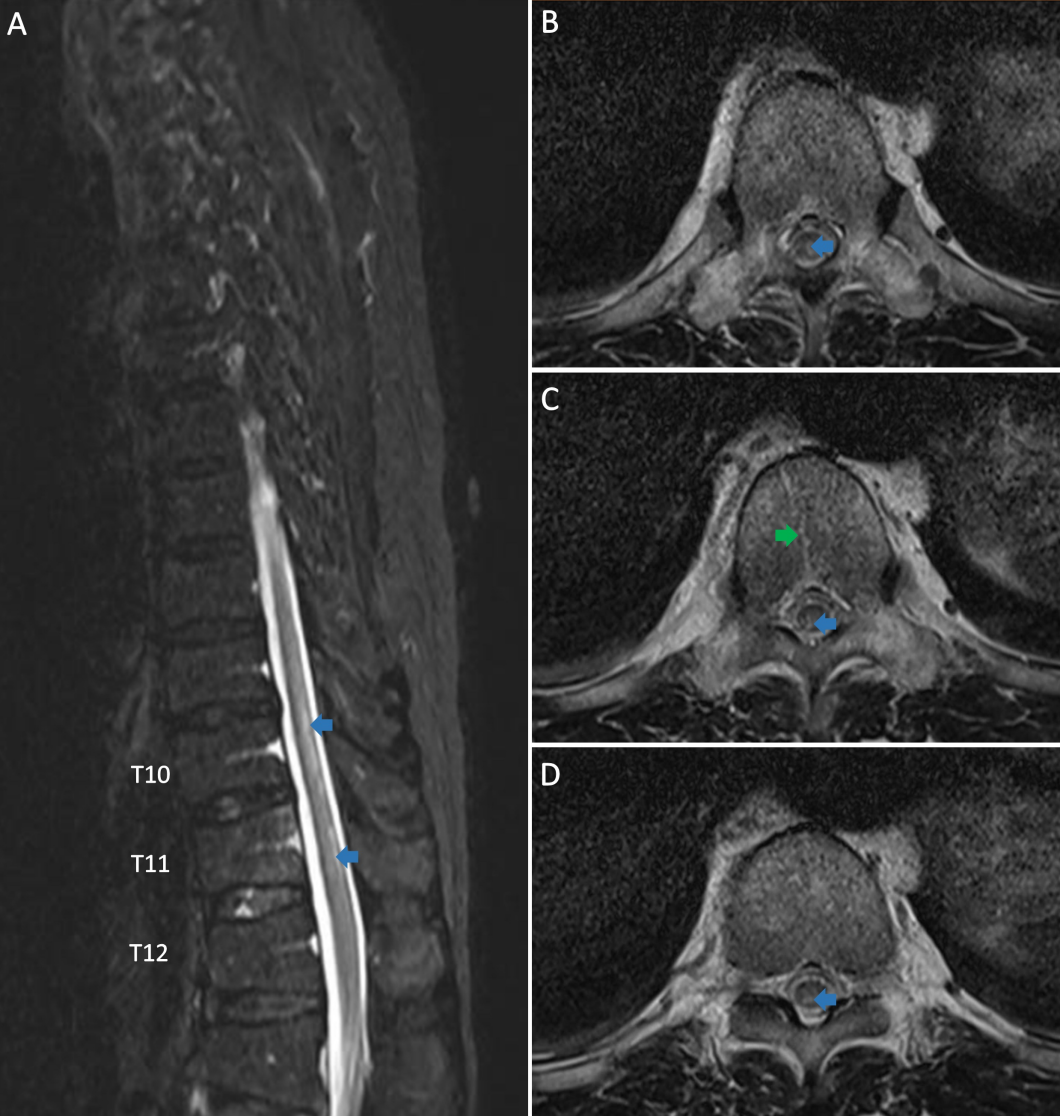
The T2-weighted magnetic resonance imaging of thoracic spine is shown, three days after symptoms onset. A: the sagittal image and B, C, D: the axial images showing hyperintensity in the posterior and posterior-central aspects of the thoracic spinal cord (blue arrows) and T2-weighted hyperintensity with enhancement is seen affecting T11 vertebra (green arrows).

The initial laboratory evaluation revealed a normal complete blood count with differential. The inflammatory markers (C-reactive protein and procalcitonin), electrolytes, liver, renal, and thyroid function tests were normal. The magnetic resonance imaging (MRI) of the thoracic spine (Figure 1) demonstrated patchy foci of the high T2-weighted signal in the posterior aspect of the lower thoracic spinal cord from the upper border of T10 to the inferior aspect of the T11 vertebral level, sparing the conus. The geographical pattern of T2-weighted hyperintensity with enhancement was seen affecting the T11 vertebra, including the posterior elements.

Based on the initial findings, the acute transverse myelitis (ATM) causing demyelination of the spinal cord was suspected and the patient was started on intravenous (IV) corticosteroid and inpatient rehabilitation. Further investigations are done to find the etiology of the ATM which was futile. The autoimmune diseases panel was negative. The cerebrospinal fluid (CSF) studies revealed white blood cell count of 18/μL (0-5) with a predominance of lymphocytes and polymorphs (no organism was seen) and the protein level of 0.65 g/L (0.1-0.4), oligoclonal bands were absent. The serum and CSF neuromyelitis optical screen was negative.

**Figure 2:**
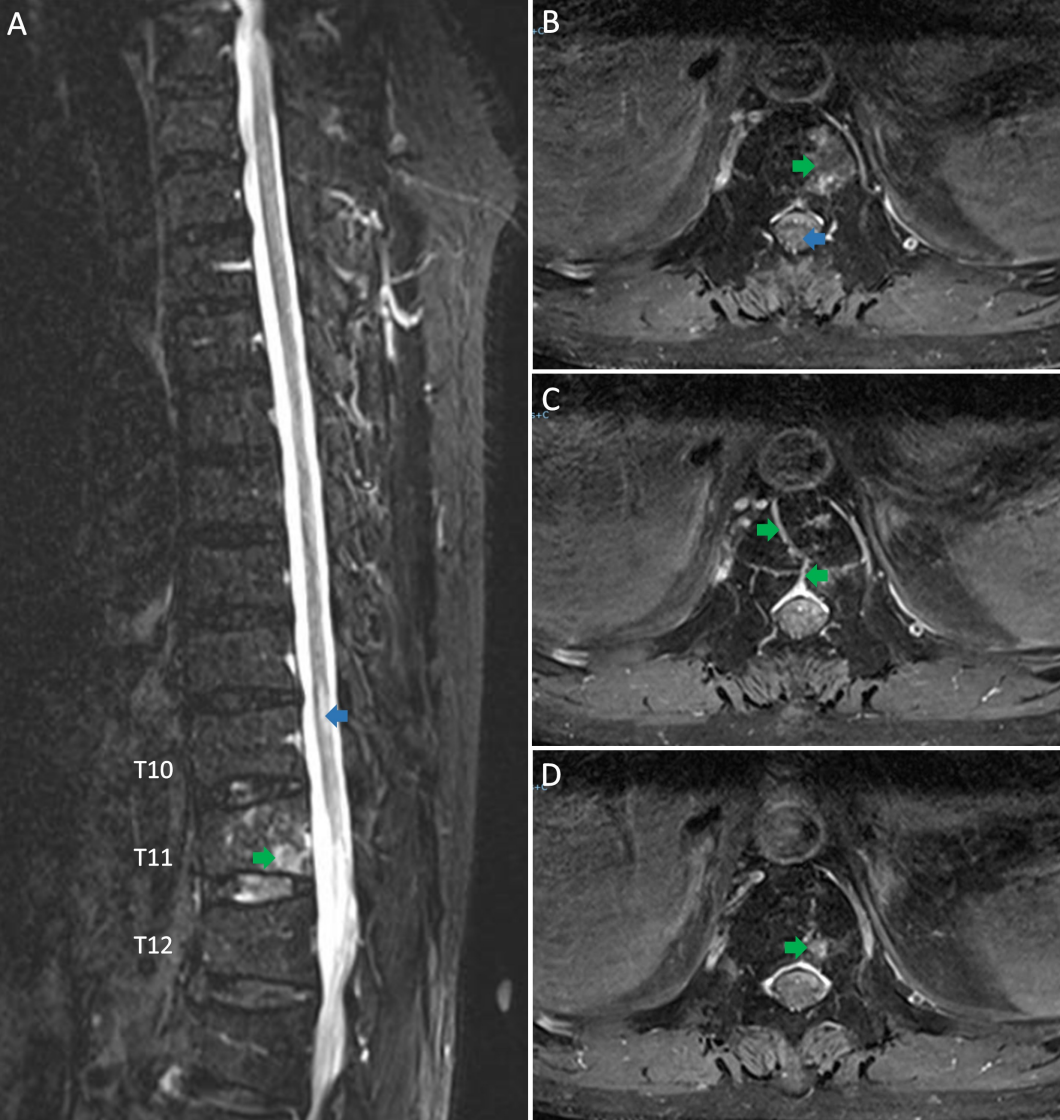
The repeat T2-weighted magnetic resonance imaging of the thoracic spine two weeks later. A: the sagittal image and B, C, D: the axial images showing central hyperintensity of the spinal cord (blue arrows) and abnormal signal within the T11 vertebral body (patchier enhancement shown by green arrow as compared to Figure 1) and the spinal cord indicated by blue arrows (less pronounced as compared to Figure 1).

Based on the noncontributory CSF analysis, the acuity of the symptoms and the presence of vertebral changes on the MRI scan, spinal cord infarction (SCI) was highly suspected. The thoracic spine MRI repeated two weeks after the initial scan (Figure 2), revealed abnormal signal within the T11 vertebral body as well as patchier enhancement. The cord signal abnormality in the lower thoracic spine was less pronounced than before and had the residual T2 signal abnormality. The diffusion-weighted images (DWI) demonstrated the faint areas of DWI hypersensitivity corresponding to the cord signal abnormalities. These evolving signal changes were highly suggestive of SCI and thus the diagnosis was established.

All the investigations (including the computed tomography of thoracic aortogram, 24-hour Holter monitor (Holter Research Laboratory, Helena Montana, United States of America), and echocardiogram) performed to elicit an etiology of SCI were futile. He was started on Clopidogrel 75mg once per day. In view of his prolonged immobility, he was started on subcutaneous enoxaparin injection 40 mg once per day but was subsequently stopped, as the patient regained assisted mobility. Inpatient rehabilitation played a pivotal role in the management. With daily physical therapy, our patient regained adequate strength in his lower limbs for home ambulation and limited community ambulation with walking frame in three months.

## Discussion

The key to diagnosing this patient was the vertebral body infarction which mimicked a vertebral fracture on the initial MRI (Figure 1). A repeat MRI of the thoracic spine revealed evolving signaling changes within the T11 vertebral body, suggesting the spinal cord and vertebral body infarction and less likely to be a vertebral fracture. This feature also differentiated it from acute transverse myelitis and other similar conditions and helped to establish the diagnosis [4-5].

The laboratory findings in SCI usually do not show any abnormalities, though the CSF protein may be elevated in some cases like in our case [1]. The MRI is the key investigation of choice [6-7]. The prompt diagnosis of the spinal cord infarction can direct towards a potentially life-threatening but treatable cause like dissection of the aorta, thrombosis or rupture of an aneurysm [2]. The MRI finding of SCI is usually normal in the acute phase of the disease (within 24 hours of symptom onset). The features of SCI emerge on MRI only after one-two days. On the other hand, nearly all the patients with transverse myelitis develop MRI features during the acute phase of the disease, a key distinguishing feature from the SCI [4-5]. Thus, it is important to repeat MRI scan in order to make the accurate diagnosis.

In our case, the first thoracic spine MRI scan was done three days after the onset of symptoms, resulting in a conflict in the diagnosis. The MRI of SCI usually demonstrates swelling and the T2 hyperintense signal of the spinal cord [2], but these findings were non-specific as transverse myelitis, neoplasms, and the inflammatory lesions can show similar characteristics [6]. However, the association of bone marrow abnormalities with spinal cord abnormalities reflects the pathology of underlying blood supply to the vertebral body which can be of diagnostic significance in SCI and can help to differentiate it from the other mimicking conditions [6].

Our patient had these symptoms when he woke up in the morning and tried to get out of bed abruptly. In contrast to cerebral arteries, the spinal arteries are more prone to mechanical damage as they run through a mobile structure [1]. The most common etiologies of SCI are aortic disease, intervertebral disc disease, spondylotic disease and spinal cord vascular malformation [1]. As in most cases, the etiology of our patient’s stroke was unknown despite an extensive evaluation [1,7].

As clinical trials were lacking, the treatment recommendations were generally based on the experiences from case series. Intravenous heparin or corticosteroid can be used during the acute phase, but the efficacy is doubtful [8]. The anticoagulation was frequently given for prevention of clot formation from immobility [8].

## Conclusions

The spinal cord infarction is a rare type of stroke and posterior spinal artery infarction is rarer. It can mimic the presentation of other spinal cord condition and can often be puzzling. The repeated MRI scan is required to make an accurate diagnosis. The vertebral body is always involved and can be of diagnostic significance as it reflects the pathology of underlying blood supply. It is important to know the exact diagnosis, as it would provide basis either for the treatment of the condition or its symptoms. Otherwise, the disease could be potentially life-threatening.
